# Overweight Patients Less Improved Kidney Function After Laparoscopic Surgery for Adrenocortical Adenoma With Excess Cortisol Secretion

**DOI:** 10.3389/fendo.2019.00572

**Published:** 2019-08-20

**Authors:** Kazuyuki Numakura, Taketoshi Nara, Sohei Kanda, Mitsuru Saito, Shintaro Narita, Takamitsu Inoue, Tomonori Habuchi

**Affiliations:** Department of Urology, Akita University Graduate School of Medicine, Akita, Japan

**Keywords:** hypercortisolism, laparoscopic adrenalectomy, kidney function, overweight, adrenal tumor

## Abstract

**Purpose:** Glucocorticoid (GC) is known to be involved in the deterioration of kidney function both directly by affecting the glomeruli and renal tubules and indirectly by affecting cardiovascular function. Autonomous GC secretion is the main feature of primary adrenal hypercortisolism (PAHC). However, the ideal treatment option (operation vs. medical treatment and observation) for patients with PAHC has not been established yet. In this study, we assessed a time series of kidney function in patients with PAHC treated via laparoscopic adrenalectomy and investigated the predictive factors for kidney function 1 year after surgery.

**Methods:** From September 1997 to July 2017, 175 laparoscopic adrenalectomies were performed for adrenal tumors at Akita University. Thirty patients, who were diagnosed as having PAHC via preoperative endocrinological evaluations and followed up for at least 1 year after surgery, were included in this study. Patients with severe complications or simultaneous aldosteronism were excluded. The mean age of the 30 patients was 57.5 years (range, 33–79 years; males, 4; females, 26), and the right and left sides were affected in 9 and 21 patients, respectively.

**Results:** In all, 18 patients were diagnosed as having Cushing's syndrome and 12 as having subclinical Cushing's syndrome. The steroid cover was required in all cases after surgery. The estimated glomerular filtration rate significantly improved (78.4 mL/min [64.8–95.8] vs. 84.1 mL/min [66.8–104.0], *p* = 0.012) 1 year after surgery. Patients showing 5% or more improvement in kidney function and those showing less than 5% improvement were compared. On performing univariate analyses, factors such as a longer operative time, heavy body mass index (BMI), and preoperative unsuppressed ACTH were associated with worse improvement in kidney function. No significant associations were observed regarding metabolic disorders, clinical symptoms, and gross proteinuria. On multivariate analysis, patients with a higher BMI (≥ 24 kg/m^2^) showed worse improvement in kidney function at 1 year after surgery (odds ratio 14.0, 95% confidence interval 1.3–142.9, *p* = 0.012).

**Conclusions:** In PAHC patients, after 1 year of follow-up, kidney function improved in terms of estimated glomerular filtration rate. Therefore, this improvement seems to be delayed in overweight patients, suggesting its direct role in renal function.

## Introduction

Primary adrenal hypercortisolism (PAHC) is characterized by autonomous cortisol secretion and is responsible for Cushing's syndrome (CS) and Subclinical Cushing's syndrome (SCS) (1). Patients with PAHC may suffer from several complications, such as obesity, hypertension, hyperglycemia, dyslipidemia, and thrombophilia (2–5). PAHC is indeed considered as an archetype of the metabolic syndrome, as insulin resistance (2, 3). Chronic glucocorticoid (GC) excess significantly affects mortality in cardiovascular disease or kidney dysfunction, causing a 2-fold to 4-fold increase in mortality compared with the general population (6). However, the ideal treatment option (operation vs. medical treatment and observation) for patients with PAHC has not been established yet. Recent studies have questioned the reversibility of complications caused by PAHC after its surgical treatment (7, 8). These findings might indicate that comorbidities such as increased cardiovascular and kidney dysfunction have negative health outcomes in patients with PAHC despite successful treatment of cortisol excess.

GC, of that almost 95% activity is consisted of cortisol, is known to be involved in the deterioration of kidney function by directly affecting glomeruli and renal tubules and indirectly affecting cardiovascular function. In fact, some clinical investigations showed the negative effect of GC on kidney function in the general population. In diabetic patients, autonomous cortisol secretion affected kidney function negatively in a dose-dependent manner (9). Serum cortisol level was measured after overnight dexamethasone suppression at a dose of 1 mg and significantly correlated with worse estimated glomerular filtration rate (eGFR) and spot urine albumin to creatinine ratio. A negative impact of cortisol on patients with essential hypertension was also reported (10). Even after adjusting for clinical factors, serum cortisol level had a negative association with the eGFR. eGFR significantly decreased with the increase in cortisol tertile.

The impact of GC excess on kidney function is less known in patients with PAHC. Haentjens P et al. reported the negative effects of GC on kidney function in CS patients and also showed that kidney dysfunction persisted even after surgery (11); however, this study was devoid of disclosing follow-up period after intervention and assessed it only in 15 patients. Other studies showed a lower creatinine clearance in patients with CS than in controls, but this difference was not statistically significant (12). However, a confirmation study concerning the implication of surgical intervention for kidney function has not been conducted in PAHC patients.

We assessed a time series of kidney function in patients with PAHC treated via laparoscopic adrenalectomy. The predictive factors for kidney function were investigated 1 year after surgery.

## Patients and Methods

### Eligibility Criteria

**Figure 1:**
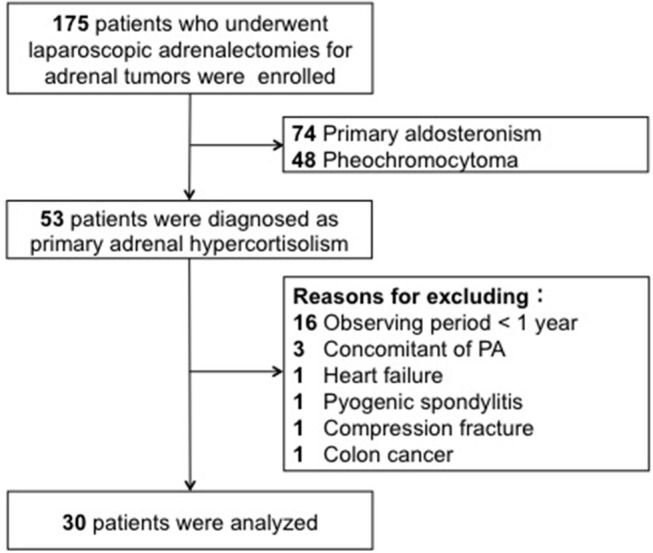
The patients who underwent laparoscopic adorenalectomy were enrolled. Of those, 53 patients were diagnosed as primary adrenal hypercortisolism. Twenty-three patients were excluded from the analysis due to a short follow-up period, severe pre-existing complications, and the simultaneous presence of aldosteronism.

We performed 175 laparoscopic adrenalectomies for adrenal tumors in Akita University from September 1997 to July 2017. Of those, 53 cases were included in this study; patients were diagnosed with PAHC on the basis of the preoperative endocrinological evaluations and followed up for at least 1 year after surgery. Exclusion criteria were simultaneous aldosteronism, major pre-existing complications, bilateral adrenal disease, or severe kidney dysfunction (preoperative eGFR <30 mL/min) (Figure 1). This study was approved by the institutional review board. All procedures were performed in accordance with the ethical standards laid down in the 1964 Declaration of Helsinki.

### Diagnostic Criteria of PAHC and ACTH Suppression

We diagnosed with PAHC in accordance with the Japan Endocrine Society Criteria (13). In the criteria, three hierarchical cortisol cutoff values, 5.0, 3.0, and 1.8 μg/dL, after a 1 mg DST was proposed. Serum cortisol ≥3 μg/dL after a 1 mg DST is to diagnose with PAHC. Serum cortisol ≥1.8–2.9 μg/dL after a 1 mg DST with a basal adrenocorticotropic hormone (ACTH) level <10 pg/mL (defined this as “ACTH suppression”) is considered to have PAHC.

### Diagnostic Criteria of Comorbidities

Diabetes mellitus: Patients with hemoglobin A1c continuously over 6.5 mg/dl, fasting plasma glucose over 126 mg/dl, or requiring insulin and/or oral hypoglycemic agents.

Dyslipidemia: Patients with high low-density lipoprotein cholesterol levels (≥140 mg/dl), hypertriglyceridemia (≥TG 150 mg/dL), and low high-density lipoprotein cholesterol levels (<40 mg/dL), or those who required oral statin treatment.

Hypertension: Patients required oral antihypertensive drugs and/or blood pressure values > 140/90 mmHg.

Psychoneurosis: Patients needed medication or had seen a psychiatrist.

Proteinuria: Positivity was assessed by a dipstick urine method (if positive, it is equivalent to over 30 mg/dL urinary protein).

### Definition of Improvement in Kidney Function

Improvement in kidney function was defined as an increase in eGFR of at least 5% from the preoperative value 1 year after surgery.

### Kidney Function

Kidney function was evaluated based on eGFR before and 1, 6, and 12 months after surgery. The eGFR was calculated using the following equation established for the Japanese population:

eGFR (mL/min/1.73 m^2^) = 194 × SCr−1.094 × Age−0.287 [× 0.739 (if female)]. The reference range of the eGFR in Japanese is ≥60 mL/min/1.73 m^2^.

### Statistical Analysis

The data were expressed as mean ± standard deviation and *p* < 0.05 was considered statistically significant. The Chi-square test was used to examine the difference in categorical data and the Mann-Whitney *U*-test was used to determine the difference in continuous values between groups. The logistic regression analysis was used for multivariate analysis. The cutoff value of continuous variables in multivariate analysis was set as the median value of each parameter. The analysis was performed using the SPSS version 24.0 statistical software (SPSS Japan Inc., Tokyo, Japan).

## Results

### Patient Characteristics

Twenty-three patients were excluded from the analysis. 16 patients had a short follow-up (<1 year). Severe pre-existing complications were observed in 4 (chronic heart failure, pyogenic spondylitis, movement difficulty due to compressed fracture, and advanced colon cancer), and the simultaneous presence of excessive secretion of aldosteronism in 3 subjects. No patient was excluded owing to severe preoperative kidney dysfunction. Of the 30 patients, 18 were diagnosed with CS and 12 with SCS. The median age of the 30 patients was 57.5 years (range, 33–79 years; males, 4; females, 26), and the right and left sides were affected in 9 and 21 patients, respectively (Table 1). Supplemental steroid treatment was required in all cases after surgery.

**Table 1:** Patients characteristics.

Sex	Male : Female	4 : 26
Age	Median year (range)	57.5 (33–79)
Laterality	Right : Left	9 : 21
Tumor diameter	Median cm (range)	3.0 (1.0–4.5)
Clinical diagnosis	CS : SCS	18 : 12
Diabetes mellitus	Yes : No	10 : 20
Hyperlipidemia	Yes : No	13 : 17
Hypertension	Yes : No	21 : 9
Psychoneurosis	Yes : No	8 : 22

*CS, Cushing's syndrome; SCS, Subclinical Cushing's syndrome*.

### Kidney Function

**Figure 2:**
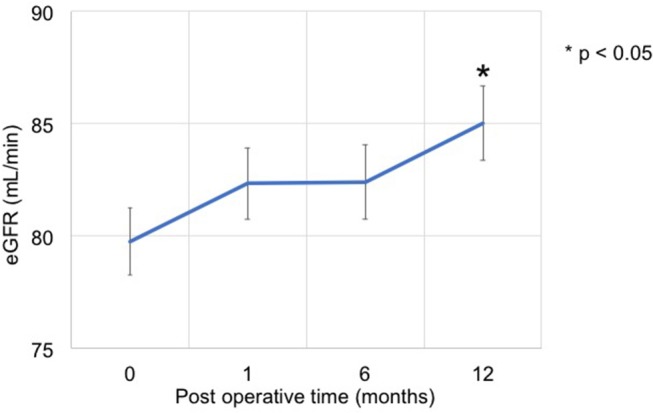
Time series of kidney function before and after adrenalectomy. The eGFR at 1 year after surgery significantly improved compared with the preoperative value (78.4 [64.8–95.8] mL/min vs. 84.1 [66.8–104.0] mL/min, *p* = 0.012).

**Figure 3:**
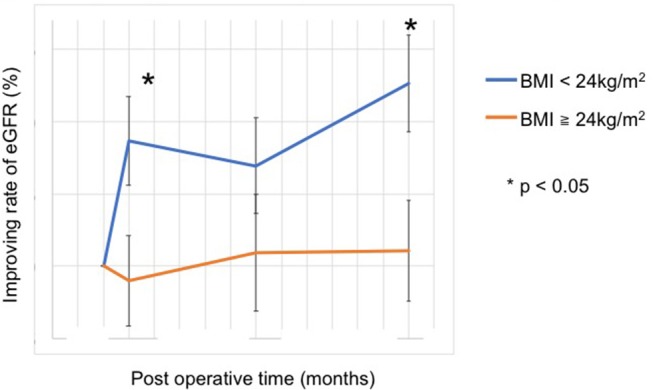
Improvement in the rate of kidney function after adrenalectomy. With a cut-off value at a BMI of 24 kg/m^2^, the improvement rate of kidney function was worse in patients with BMI ≥ 24 kg/m^2^ at 1 month and 1 year after surgery (1 month, 8.6 [−6.2–30.4]% vs. −0.9 [−18.9–22.1]%, *p* = 0.038; 1 year, 12.6 [−14.0–42.4]% vs. 1.1 [−22.2–21.3]%, *p* = 0.024).

The eGFR significantly improved 1 year after surgery compared with the preoperative value (78.4 [64.8–95.8] mL/min vs. 84.1 [66.8–104.0] mL/min, *p* = 0.012) (Figure 2); however, no significant improvement was observed at 1 and 6 months. Patients showing 5% or more improvement in kidney function and those showing <5% improvement were compared. On univariate analysis, factors such as left adrenal mass, increased body mass index (BMI), and the presence of preoperative unsuppressed ACTH were associated with worse improvement in kidney function (Tables 2, 3). On multivariate analysis, patients with a higher BMI (≥ 24 kg/m^2^) showed a worse improvement rate in kidney function 1 year after surgery (odds ratio 14.01, 95% confidence interval 1.30–142.86, *p* = 0.012) (Table 4). When the patients were divided into two groups according to the BMI, with a cutoff point at 24 kg/m^2^, the improvement in kidney function in the BMI ≥ 24 kg/m^2^ group was < that in the BMI <24 kg/m^2^ group 1 month and 1 year after surgery (1 month; −0.9 [−18.9–22.1]% vs. 8.6 [−6.2–30.4]%, *p* = 0.038: 1 year; 1.1 [−22.2–21.3]% vs. 12.6 [−14.0–42.4]%, *p* = 0.024) (Figure 3).

**Table 2:** Clinical predictive factors for improvement in kidney function after surgery (continuous variable).

	**Median (range)**	**Improvement (*n* = 17)**	**Less-improvement (*n* = 13)**	***p***
Age	Year	57.0 (34.0–73.0)	59.0 (33.0–79.5)	0.805
Cortisol	μg/dL	12.2 (5.1–41.2)	15.1 (6.6–48.1)	0.281
ACTH	pg/mL	2.0 (<2.0–8.9)	4.5 (<2.0–34.0)	0.145
Cortisol after 1 mg DST*	μg/dL	9.3 (1.8–32.7)	11.8 (3.4–34.0)	0.646
BMI	kg/m^2^	23.5 (17.6–28.0)	26.4 (20.3–44.4)	0.014
Pre operative serum creatinine	mg/dL	0.56 (0.41–0.97)	0.60 (0.40–1.08)	0.170
Pre operative eGFR	mL/min/1.73 m^2^	77.2 (38.8–119.8)	79.7 (40.2–120.5)	0.869
Steroid supplement duration	Month	13.0 (2.0–72.0)	14.5 (1.0–104.0)	0.867
Tumor diameter	cm	3.0 (1.0–4.0)	3.0 (1.6–4.5)	0.650

ACTH, adrenocorticotropic hormone; DST, dexamethasone suppression test; BMI, body mass index; eGFR, estimated glomerular filtration rate.

* *The actual cortisol value after 1 mg DST were missing in four patients*.

**Table 3:** Clinical predictive factors for improvement in kidney function after surgery (categorical variable).

		**Improvement (*n* = 17)**	**Less-improvement (*n* = 13)**	***p***
Sex	Male : Female	1 : 16	3 : 10	0.290
Laterality	Right : Left	8 : 9	1 : 12	0.042
1 mg DST	Positive : Negative	15 : 2	11 : 2	0.800
ACTH suppression	Positive : Negative	17 : 0	9 : 4	0.026
Urine protein	Positive : Negative	3 : 14	6 : 7	0.198
Clinical diagnosis	CS : SCS	11 : 6	7 : 6	0.711
**Comorbidities**				
Diabetes mellitus	Yes : No	7 : 10	3 : 10	0.515
Hyperlipidemia	Yes : No	8 : 9	6 : 7	0.749
Hypertension	Yes : No	11 : 6	10 : 3	0.748
Psychoneurosis	Yes : No	3 : 14	5 : 8	0.389
**Symptoms**				
Central obesity	Yes : No	6 : 11	6 : 7	0.821
Moon face	Yes : No	8 : 9	8 : 5	0.676
Buffalo hump	Yes : No	3 : 4	7 : 2	0.152
Skin thinning	Yes : No	1 : 4	4 : 1	0.058
Striae cutis	Yes : No	3 : 5	6 : 3	0.229
Polytrichosis	Yes : No	1 : 3	3 : 3	0.429
Acne	Yes : No	1 : 0	5 : 1	0.659
Amenorrhea	Yes : No	2 : 3	3 : 0	0.090

*DST, dexamethasone suppression test; ACTH, adrenocorticotropic hormone; CS, Cushing's syndrome; SCS, Subclinical Cushing's syndrome*.

**Table 4:** Multivariate analysis of risk factors for delayed improvement in kidney function.

**Factor**	**Risk category**	**Improvement (*n* = 17)**	**Non-improvement (*n* = 13)**	**Univariate**			**Multivariate**		
				**Odd's ratio**	**95% CI**	***p***	**Odd's ratio**	**95% CI**	***p***
Laterality	Left	9: 8	12: 1	2.07	1.20–3.58	0.042	3.41	0.26–45.45	0.335
Pre-operative ACTH suppression	Negative	17: 0	9: 4	2.89	1.70–4.90	0.026	-	-	1.000
BMI (kg/m^2^)	≥24	12: 5	3: 10	2.40	1.12–5.13	0.025	14.01	1.30–142.86	0.012

*ACTH, adrenocorticotropic hormone; BMI, body mass index; CI, confidence interval*.

## Discussion

The results of this study showed an improvement in eGFR after adrenalectomy in patients with PAHC. In this study, eGFR increased from 78.4 mL/min/1.73 m^2^ to 84.1 mL/min/1.73 m^2^ (*p* = 0.012) after surgery. A high BMI (≥24 kg/m^2^) was an independent predictor of worse improvement rate in eGFR after surgery.

GC is known to cause deterioration in kidney function both directly by affecting glomerular and tubular function and indirectly by affecting the cardiovascular system (14). Excess GC due to endogenous GC overproduction in PAHC plays a pivotal role in hypertension and causes increased cardiac output, total peripheral resistance, and renal blood flow (9). Focusing on the effect of GC on the kidney, GCs increased renal vascular resistance in the general population (15), and chronic GC exposure may decrease GFR and lead to a higher cardiovascular risk in patients with PAHC (14). However, little is known about kidney function after adrenalectomy in PAHC (7, 12, 16). In this study, 15 patients (50%) showed more than 5% improvement in eGFR 1 year after surgery without regard to the prevalence of hypertension or other metabolic disorders. Moreover, morbidity with metabolic complication did not affect kidney function (Table 3). This result might indicate that kidney dysfunction caused by cortisol exposure was reversible. Although kidney function after surgery has not yet been investigated (11, 12), the reversibility of urinary albumin excretion (UAE) by normalizing cortisolemia was reported (16). As is well-known, UAE is used as a preclinical sign of kidney injury. The median eGFR immediately before surgery was 78.4 mL/min in our series, and this value was relatively better than that in the age-matched general Japanese population (17). In other words, patients with PAHC in our series might less suffer from kidney injury before intervention and then showed marked increasing kidney function.

Overweight was an independent risk factor for delayed improvement in kidney function in our study. Both indirect and direct mechanisms of the negative impact of obesity or overweight on kidney function can be explained. Kidney function independently improved following weight loss without affection by comorbidities after bariatric surgery (18). Adipose tissue mass negatively affects kidney function (19), and increasing muscle mass protects against renal injury in the obese population (20). GC augments adipose tissue and deteriorates skeletal muscle through its catabolic effects (21). Normalizing the GC level could reduce adipose tissue mass and ameliorate skeletal muscle (22), thus leading to an improvement in kidney function. An indirect mechanism was explained by a different isoform of 11β-HSD, which is the main metabolic enzyme in the GC metabolic pathway and has two different isoforms (23). One of them, 11β-HSD1, is a dominant isoform in obese individuals and the other isoform, 11β-HSD2, is less active. An aberration of 11β-HSD1 resulted in a high plasma level of GC, and its prolongation might cause irreversible organ injury, especially in the kidney (24).

Practically, there is a debate regarding whether a BMI of 24 kg/m^2^ is overweight or not. Undoubtedly, a BMI of 24 kg/m^2^ is not obese or overweight in international consensus. However, a BMI of 25 kg/m^2^ or higher would be regarded as overweight in Japan (25), but the ideal cut-off value of BMI varies depending on the ethnicity (26). Moreover, BMI might already be an impractical marker to predict individual adipose tissue mass. Other precise markers and concepts have been proposed and have gained greater prominence instead of BMI, such as diagnostic scores in sarcopenia obesity (27, 28). Indeed, determination of a valid cutoff value of BMI is difficult. Therefore, further verification is necessary.

Several limitations of this study should be noted, including the retrospective nature and the limited number of patients. A relatively high number of patients were excluded because of the short follow-up period. This might influence the study results. However, our results show promising relevance for surgical treatment and warrant further research on kidney function in patients with PAHC.

## Conclusions

This study demonstrated the favorable outcomes of kidney function via laparoscopic adrenalectomy in patients with PAHC. Although PAHC patients have multiple metabolic disorders, kidney function was reversible. Our findings suggest that adrenalectomy is a reasonable treatment option for patients with PAHC. Future prospective studies with larger sample sizes are required to better understand the appropriate management for patients with PAHC.

## Ethics Statement

Human participants: Research involving human participants was supervised and approved by the Ethics Board of the Akita University Hospital.

## Informed Consent

All the participants in this study provided written informed consent.

## Author Contributions

KN: project development, data collection and management, data analysis, patient management, and manuscript writing. TN, SK, MS, SN, and TI: patient management. TH: project development, management, and manuscript editing.

### Conflict of Interest Statement

TH received honoraria from Novartis Pharm, Pfizer Co, GlaxoSmithKline, and Ono Pharma Co. The remaining authors declare that the research was conducted in the absence of any commercial or financial relationships that could be construed as a potential conflict of interest.
